# Neutrophil Extracellular Trap Formation: Physiology, Pathology, and Pharmacology

**DOI:** 10.3390/biom9080365

**Published:** 2019-08-14

**Authors:** Mithunan Ravindran, Meraj A. Khan, Nades Palaniyar

**Affiliations:** 1 Program in Translational Medicine, SickKids Research Institute, The Hospital for Sick Children, Toronto, ON M5G1X8, Canada; 2 Faculty of Medicine, University of Toronto, Toronto, ON M5S1A8, Canada; 3 Department of Laboratory Medicine and Pathobiology, University of Toronto, Toronto, ON M5S1A8, Canada; 4 Institute of Medical Sciences, University of Toronto, Toronto, ON M5S1A8, Canada

**Keywords:** Neutrophil extracellular traps, Nox-dependent/Nox-independent/suicidal/vital NETs, cystic fibrosis, autoimmunity, diabetes, cancer metastasis

## Abstract

Neutrophil extracellular traps (NETs), a unique DNA framework decorated with antimicrobial peptides, have been in the scientific limelight for their role in a variety of pathologies ranging from cystic fibrosis to cancer. The formation of NETs, as well as relevant regulatory mechanisms, physiological factors, and pharmacological agents have not been systematically discussed in the context of their beneficial and pathological aspects. Novel forms of NET formation including vital NET formation continue to be uncovered, however, there remain fundamental questions around established mechanisms such as NADPH-oxidase (Nox)-dependent and Nox-independent NET formation. Whether NET formation takes place in the tissue versus the bloodstream, internal factors (e.g. reactive oxygen species (ROS) production and transcription factor activation), and external factors (e.g. alkaline pH and hypertonic conditions), have all been demonstrated to influence specific NET pathways. Elements of neutrophil biology such as transcription and mitochondria, which were previously of unknown significance, have been identified as critical mediators of NET formation through facilitating chromatin decondensation and generating ROS, respectively. While promising therapeutics inhibiting ROS, transcription, and gasdermin D are being investigated, neutrophil phagocytosis plays a critical role in host defense and any therapies targeting NET formation must avoid impairing the physiological functions of these cells. This review summarizes what is known in the many domains of NET research, highlights the most relevant challenges in the field, and inspires new questions that can bring us closer to a unified model of NET formation.

## 1. Introduction

**Figure 1:**
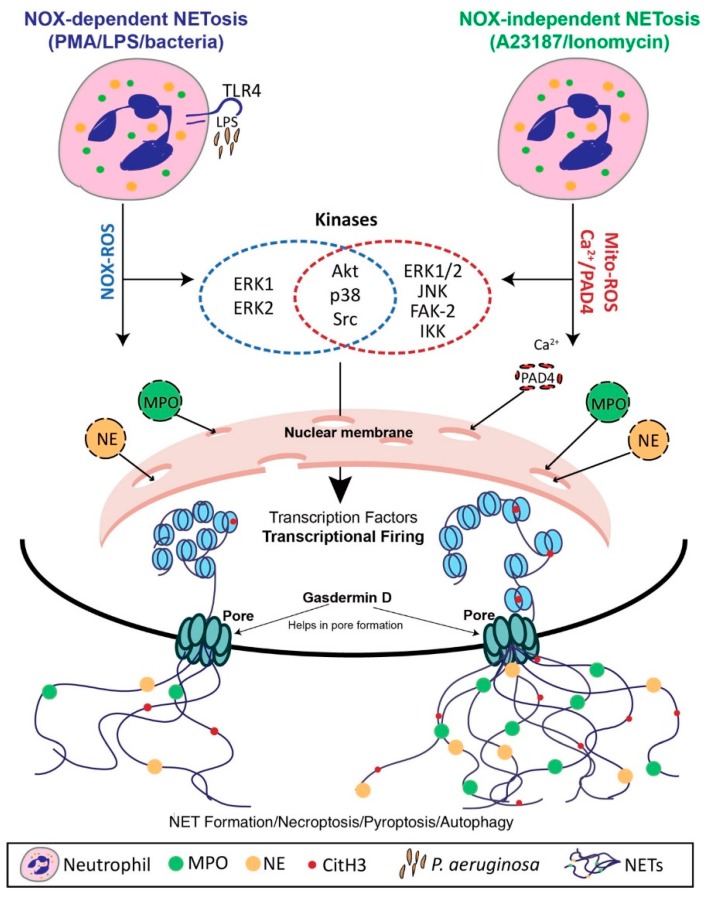
A unified model of neutrophil extracellular trap (NET) formation. Different agonists induce either Nox-dependent (agonists: Phorbol 12-myristate 13-acetate (PMA), LPS, bacteria etc.) or Nox-independent NET formation (agonists: A23128, ionomycin, uric acid crystals etc.). These agonists induce different forms of reactive oxygen species (ROS) (Nox-ROS and Mitochondrial-ROS). Both of these ROS activate different sets of kinases specific to Nox-dependent or Nox-independent NET formation. These kinases then activate transcription factors leading to transcriptional firing at promotor regions. This process enables chromatin decondensation and is further facilitated by PAD4-mediated citrullination in Nox-independent NET formation. Enzymes such as neutrophil elastase and myeloperoxidase enter the nucleus and decorate the chromatin. Finally, in certain forms of NET formation, the nucleus disintegrates, and NETs are released, whereas in others the protein gasdermin-D helps to form pores to enable the release of NETs.

Due to their unique role in innate immunity and a variety of pathological processes, neutrophil extracellular traps (NETs) have become the subject of much investigation in the last decade. The pace at which the mechanistic details of established processes are being elucidated, including the role of transcription in both NADPH (Nox)-dependent and independent NET formation, and various forms of vital NET formation, is rapid [1,2]. For this reason, we feel that it is essential to review the landmark discoveries in NET research as a foundation while also discussing contentious concepts, including the role of protein arginine deiminase 4 (PAD4) in Nox-dependent NET formation [3,4,5,6]. Many new details about the context dependency of NET formation have emerged, demonstrating how this process differs in the bloodstream versus in the tissue and under alkaline or hypertonic conditions [7,8,9]. In this review, we will highlight how and why NETs have been implicated in the pathogenesis of common and deadly diseases, ranging from atherosclerosis to cancer [10,11]. This will inform our discussion of the mechanisms and potential efficacy of the latest NET-based therapeutics including gasdermin D inhibitors, transcription inhibitors, and DNase administration [1,12,13]. Our purpose is to provide a succinct, yet comprehensive, overview of the latest updates in the field of NET formation research. A potential unified model of NET formation has been updated in Figure 1.

## 2. The Discovery of NET Formation

For several decades, neutrophils were thought to die primarily by apoptosis or necrosis, and, more recently, necroptosis [14]. In 1996, Takei et al. discovered an interesting novel form of neutrophil death that was neither apoptosis nor necrosis [15]. When the authors stimulated neutrophils with phorbol 12-myristate 13-acetate (PMA), these cells died by decondensing their chromatin, dissolving their nuclear membranes, and releasing chromatin coated with granular proteins [15]. In 2004, this finding was confirmed and extended to include other agonists, such as IL-8 and LPS, and NETs were considered to be an extracellular anti-bacterial structure [16]. Initial studies indicated that NETs form within minutes of stimulation and the neutrophils were not considered dead [16]. However, the same lab in 2007 showed that only dying neutrophils released NETs between 3–4 h [17]. Studies conducted by several other labs also showed that the majority of the neutrophils release NETs while dying 2–4 h after activation [1,3,18,19,20]. This suicidal form of NET formation was initially considered to be NADPH-oxidase (Nox)-dependent [17,21]. However, in recent years our group and others have demonstrated that Nox-independent NET formation is facilitated through calcium influx and mitochondrial ROS production, as we will discuss in further detail [3]. These earlier studies raised the question of whether neutrophils could function in the absence of their DNA.

Interestingly, in the 1980s, Malawista and colleagues showed that enucleated neutrophils, termed cytoplasts, retained chemotactic and antibacterial properties, indicating that neutrophil DNA is not essential for neutrophil survival and some of their key functions [22]. This study demonstrated that it was indeed possible for vital NET formation to occur. Three different types of vital NET formation have also been described to date. In 2009, Yousefi et al. showed that in response to the complement component C5a and GM-CSF or LPS, neutrophils release mitochondrial DNA as extracellular traps [2]. This form of vital NET formation is akin to eosinophils releasing mitochondrial DNA as catapult-like extracellular structures [23]. In 2010, Pilsczek et al. showed that Panton Valentine toxin of *Staphylococcus aureus* could induce NOX-independent vital NET formation that generate nuclear blebs that are released as NETs [23,24]. Additionally, a form of vital NET formation dependent upon platelet surface TLR4 responding to lipopolysaccharide in the cell walls of Gram-negative bacteria has also been described [25,26].

Another set of studies have also identified the importance of histone modification in NET formation, which we will also further elucidate in this review [5,27,28]. It has been demonstrated that, in addition to superoxide production, PMA and LPS-induced NET formation require autophagy [21,29]. Remijsen et al. (2011) demonstrated this through the administration of a PI3K inhibitor, an inhibitor of autophagy, which subsequently also inhibited NET formation [21]. Beyond this, the work of McInturff et al. (2012) has revealed that rapamycin can regulate NET formation through hypoxia-inducible factor, a well-known mediator of autophagy [29,30]. Indeed, it has even been noted recently that UV rays are capable of inducing a unique form Nox-independent NET formation that involves both apoptosis and NET formation simultaneously known as ApoNETosis, though this interesting development requires further investigation [31]. These advancements demonstrated that much has changed since the initial discovery of NETs and that much investigation remains to fully elucidate their role in physiological and pathological processes.

While the term “NETosis” is commonly used, concerns about its appropriateness have been raised in light of findings, such as the different forms of vital NET formation, thus far described [32,33]. There is, therefore, compelling evidence that NETs can be produced in the absence of cell death, an important finding that can be lost when describing this process as “NETosis” [2,25]. In particular, the Nomenclature Committee on Cell Death (NCCD) in 2018 recommends that the term “NETosis” be avoided, in the absence of evidence of cell death [32]. This represents an important shift in terminology but also a significant challenge because some groups such as Yipp et al. have coined terms including “vital NETosis” to describe their work and other groups citing this work have continued to use this term [25]. Other groups such as Yousefi et al. refer to NET extrusion in the absence of cell death as “NET formation” in the context of cell death so there is not yet consistency across the field [2,33]. For the purposes of this review, we will follow the NCCD recommendations and use the term “NET formation” rather than “NETosis”. We recognize that the use of the term “NETosis” is currently an area of contention and one likely to change in the near future.

## 3. The Role of NET Formation in Complement Activation

Activation of the complement system occurs via three mechanisms; the classical, lectin, and alternative pathways (AP). Our group has demonstrated that neutrophils stimulated with PMA, anaphylatoxin C5a, and formyl–methionyl–leucyl–phenylalanine (fMLP) release essential AP components including C3, complement factor P (CFP), complement factor B (CFB) [34]. These three AP components are necessary for the assembly of the convertase C3bBb which goes on to cleave C3 into C3a and C3b [35]. C3b binds to C3bBb and forms C3bBbC3b, C5 convertase, which is necessary for the formation of the membrane attack complex (C5b-9) [35]. C5b-9 was found to be deposited on NETs during NET formation and this effect was abrogated through the use of DNase, indicating the importance of these structures in the formation of the membrane attack complex in this specific context [34]. Our findings are in line with previously published work, demonstrating that complement proteins deposit on NETs and that NET formation can induce AP complement activation in response to Gram-negative bacterial infections and anti-neutrophil cytoplasmic antibody (ANCA)-associated vasculitis (AAV) [36,37,38]. These results have important implications for other NET-associated pathologies, such as systemic lupus erythematosus (SLE), rheumatoid arthritis (RA), and cystic fibrosis (CF), especially considering that complement inhibitors such as eculizumab has been investigated in other contexts such as atypical hemolytic uremic syndrome [39]. 

## 4. Mechanisms of NET Formation

### 4.1. Nox-Dependent NET Formation

Of the various NET formation mechanisms known today, Nox-dependent NET formation is the best described. Agonists such as PMA and LPS have been well-validated in the literature, however, studies including our own have demonstrated that they induce Nox-dependent NET formation through two different mechanisms [16,40]. Following the entry of PMA, a diacylglycerol mimetic, endoplasmic reticulum sources of calcium enter the cytosol where they increase the activity of protein kinase C, which then phosphorylates gp91phox/Nox2 [41]. There is contention as to whether this form of intracellular calcium increase is insufficient to activate PAD4 and citrullination of histones [3,5,6]. This issue will be explored in further detail in this section. Regardless, this process facilitates the assembly of the Nox enzyme, thereby driving the generation of ROS, which have been documented to subsequently disintegrate the membranes of granules and the nucleus alike [17,42]. While normally contained within azurophilic granules, neutrophil elastase (NE) and myeloperoxidase (MPO) are now free to interact with the nucleus where they may cleave histones and facilitate chromatin decondensation [42]. This process culminates in loss of membrane integrity of the neutrophil and the decondensed DNA, which by this point is decorated with granular contents, is released into the extracellular milieu to carry out anti-microbial functions [16,42]. It should be noted that PMA, though used ubiquitously, is a non-physiological Nox-dependent agonist, so such experiments investigating the mechanism of NET production and their role in disease should be considered carefully. Conversely, LPS is commonly found in the context of infection by Gram-negative bacteria and so may offer more utility for experimental models that aim to more closely mimic in vivo conditions. 

Our group found that LPS-induced Nox-dependent NET formation functions through a separate pathway mediated by c-Jun N-terminal kinases (JNK) [43]. While the mechanism is similar to PMA following phosphorylation of Nox2, upstream of this step there are important differences [43]. We determined that LPS binding to TLR4 on the neutrophil surface induces dose-dependent Nox-dependent NET formation and that inhibition using TAK242 abolishes this effect [43]. Furthermore, JNK inhibitors such as SP600125 and TCSJNK6o significantly interfered with LPS-induced NET formation but not PMA, indicating that these two agonists do indeed elicit their effects through two different mechanisms [43]. 

The role of PAD4 in Nox-dependent NET formation remains an area of contention. Our group and others have previously demonstrated that PAD4, while required for Nox-independent NET formation, does not play an integral part in Nox-dependent NET formation [3,4]. This was shown through confocal microscopy, which found that upon stimulation with PMA, NET formation occurred without detectable histone deimination [3,4,44]. However, there has been contrary evidence presented that indicates that LPS and PMA-mediated NET formation may indeed require PAD4 [5,6]. Evidence in support of this position include findings that PAD4 knockout murine neutrophils are unable to produce NETs in response to PMA or LPS stimulation and inhibition of PMA-mediated NET formation through the administration of PAD4 inhibitors such as chloraminidine GSK484 [5,6]. 

### 4.2. Nox-Independent NET Formation

The relevance of Nox-independent NET formation was highlighted by the work of Parker et al. (2012), which suggested that calcium ionophores, such as ionomycin, could induce the formation of NETs in a Nox-independent manner [45]. Similar to PMA, these agonists are non-physiological and experiments in which they are involved should be considered with this caveat in mind. Large amounts of PAD4 are already present in the cytosol and once bound with the calcium from the influx facilitated by relevant ionophores, translocate into the neutrophil nucleus. Here the enzyme deiminates histone arginine residues carrying a positive charge into neutral citrulline [28]. This has been suggested to result in chromatin decondensation necessary for Nox-independent NET formation [28]. We found hypercitrullination of histone H3 in NET formation mediated by Nox-independent agonists but not Nox-dependent agonists, confirming the relevance of citrullination of histones in NET formation [3,4]. This concept is further supported by findings that while A23187, a Nox-independent NET formation agonist, induces histone H3 citrullination, PMA, a Nox-dependent NET formation agonist, does not [44]. Citrullination of promoters is of particular importance since this provides access to transcription factors we have previously demonstrated to be necessary for NET formation [1]. 

Our group has further provided pieces of the puzzle in the form of the role of the calcium-activated potassium channel of small conductance (SK channel) and mitochondrial ROS (mROS) [3]. By observing a reduction in Nox-independent NET formation following SK3, the most commonly expressed SK channel on neutrophils, knockdown and induction following treatment with 1-Ethyl-2-benzimidazolinone (EBIO), a SK channel-specific activator, we verified the necessity of SK channels in this process [3]. Additionally, incubation with dinitrophenol (DNP), a mitochondrial ATP production uncoupler, inhibited mROS production leading to a significant and dose-dependent reduction in Nox-independent NET formation [3].

Attributing an important role in Nox-independent NET formation to mitochondria is a significant finding because these organelles play a minimal role in ATP synthesis in neutrophils, which begs the question of what function do they serve [46]. mROS produced through activation of the SK channel had previously been attributed to apoptosis but we now contend that this process also plays a role in innate immunity and, potentially, pathology [3,47]. Therefore, we have assigned a novel role for mitochondria in neutrophils, to serve as a ROS generator and facilitate the innate immune function of neutrophils via Nox-independent NET formation.

### 4.3. The Role of Transcriptional Firing in NET Formation

Given that inactivated neutrophils only survive for a matter of hours, the role of transcription in these cells remained a mystery for many years because there is little need for them to conduct de novo protein synthesis [1,48,49,50]. Faced with this puzzle, our group assigned a potential function to transcriptional firing as a necessary step in both Nox-dependent and independent NET formation [1]. In particular, it was noted that transcriptional firing at promoter regions played a role in promoting the DNA decondensation that is required for NET formation [1]. Both forms of NET formation are suppressed by blockade of transcription but not translation and the inhibition of transcription did not impair ROS generation for ordinary antimicrobial functions, a finding with important therapeutic implications [1]. We further elucidated the two mechanisms of Nox-dependent and independent NET formation by identifying certain kinase cascades and transcription factors that predominate in one pathway or another [1,19]. For example, transcription of Erk, Akt, p38, and cSrc-regulated genes is the primary driver of Nox-dependent NET formation, whereas Akt, p38, cSrc, PyK2 and Jnk-regulated genes predominate in Nox-independent NET formation [1]. 

There remains controversy with regards to the role of transcription in NET production given conflicting evidence as to whether it is an integral mechanistic component [1,50]. Sollberger et al. (2016) previously demonstrated that transcription is not integral to the PMA-induced production of NETs by finding that the transcription inhibitors actinomycin D, flavopiridol, and CAS 577784-91-9 did not suppress NET formation [50]. Similarly, Tatsiy and McDonald (2018) found that actinomycin D did not suppress NET formation [6]. We, however, demonstrated previously that actinomycin D did indeed inhibit both PMA and A231287-induced NET formation in a dose-dependent manner [1]. It is important to note, however, that Sollberger et al. (2016) and Tatsiy and McDonald (2018) both conducted their experiments under serum conditions while we did not. This difference in experimental conditions could explain how the effect of inhibiting transcription may be dependent on the physiological context of NET formation. Therefore, further investigation to account for this difference in experimental conditions is required before more definitive conclusions can be drawn about the role of transcription in NET formation and how these findings can be used to inform the development of novel therapeutics. 

### 4.4. Vital NET Formation

Another form of NET formation, distinct from those discussed thus far, has also emerged in which neutrophils are able to survive expelling NETs and continue their antimicrobial function [25,26]. This process occurs much faster than suicidal NETs, occurring in just 5–60 min, and comes in two flavors involving either nuclear or mitochondrial DNA [2,25,26]. Nuclear DNA vital NET formation, proposed by Yipp and Kubes (2013) relies on the binding of LPS, in the case of Gram-negative bacteria, to TLR4 on the surface of platelets and the resulting crosstalk with neutrophils produces NETs in a Nox-independent manner [25,26]. The response to Gram-positive bacteria requires both toll-like receptor 2 and complement receptor 3. To demonstrate that this form of NET formation is indeed vital, Yipp and Kubes employed intravital microscopy to visualize neutrophils in the context of a mouse skin infection [25,26]. These neutrophils were still able to undergo chemotaxis and phagocytose bacteria following the release of NETs. Yousefi et al. (2009) have proposed that mitochondrial DNA vital NET formation requires both GM-CSF and LPS or C5a [2]. As the name suggests, the primary difference between this form of NET formation and the one proposed by Yipp and Kubes is that the DNA detected by Yousefi et al. was mitochondrial, rather than nuclear, and requires mitochondrial ROS. This vital process has been demonstrated to be Nox-dependent, in contrast to a similar mitochondrial ROS-dependent innate immune process known as basophil extracellular traps (BETs), which are Nox-independent [2,23,51]. 

### 4.5. The Role of Histone Modifications in NET Formation

As mentioned previously, there remains some controversy around whether histone H3 deimination by PAD4 is required for both Nox-dependent and Nox-independent NET formation, however, this is not the only form of histone modification that is relevant to NET formation [3,4,52,53]. Histone methylation, through catalysis by histone methyltransferase, involves the transfer of a methyl group from S-adenosyl methionine to histones. There have not yet been studies investigating the specific role of histone methylation in NET formation, a role for this process has been suggested indirectly [52]. It has been shown that PAD4 may be able to regulate methylation of H3 and H4 arginine residues in vivo by converting methylated arginine to citrulline. Given that histone methylation promotes condensation, the opposite of what is required in NET formation, further investigation of this finding may yield a new therapeutic locus of interference [52,54,55]. 

Our group has also previously demonstrated that histone acetylation promotes NET formation [53]. The biological plausibility of this finding is supported by the literature, which demonstrates that this process neutralizes the positive charge on the histone, thereby impairing the interaction with the negatively charged DNA and promoting the decondensation necessary for NET formation [56]. Therefore, histone deacetylase complexes (HDAC) would promote DNA condensation [57]. Using HDAC inhibitors, we demonstrated that increasing histone acetylation promotes NET formation at baseline, as well as when induced by Nox-dependent and Nox-independent NET formation [53]. Furthermore, we found that HDAC inhibitors have a biphasic effect on NET formation, specifically that at lower doses agents such as belinostat promote NET formation dose-dependently (0–0.25 µM) but at higher doses (>1 µM) inhibit this effect [58]. This new finding, while exciting, requires further corroboration with other groups but presents yet another new avenue of investigation by knocking down HDAC to potentially inhibit NET formation. 

## 5. NET Formation in Various Physiological Contexts

It is necessary to understand the various known mechanisms of NET formation in order to appreciate how this process differs in different physiological contexts, including in the bloodstream or tissue and under alkaline or hypertonic conditions. Bloodstream, LPS-induced NET formation occurs in less than an hour through a vital, platelet-dependent, and Nox-independent process [7,59]. LPS-induced NET formation in peripheral tissues takes place over 2-4 h and is a suicidal, Nox-dependent process [7]. While *E. coli* LPS serotypes *O55:B5, O127:B8, O128:B12, O111:B4*, and *O26:B6*, as well as LPS from *Salmonella enterica* (serotype *enteritidis*), and *Pseudomonas aeruginosa* (serotype 10) were all able to induce NET formation under intravascular conditions, only *O128:B12* and *Pseudomonas aeruginosa* (serotype 10) were able to do so under tissue conditions [7]. This indicates that neutrophils can selectively respond to LPS serotypes depending on the context [7]. These discrepancies help explain how the NET response is able to simultaneously mount a vigorous response to life-threatening intravascular infections while avoiding an overzealous response to a potentially more manageable tissue infection, thereby avoiding unnecessary damage to the host.

Our group has also demonstrated that Nox-independent NET formation is promoted by a more alkaline pH [8]. It was found that with increasing environmental pH, neutrophils stimulated by A23187 and ionomycin had a significantly greater rise in intracellular pH as compared to unstimulated cells [8]. This is significant because intracellular calcium, mitochondrial ROS, and PAD4-mediated citrullination of histones are all promoted by alkaline pH and, as has been previously discussed, these are all integral elements of the Nox-independent NET formation pathway [8]. The biological plausibility of these findings are supported by the notion that enzymes such as PAD4 and NE, crucial enzymes in Nox-independent NET formation, have an alkaline pH optimum. We similarly found that Nox-dependent NET formation is promoted by alkaline pH [60]. In this case, ROS production was elevated at higher pH, as was histone H4 cleavage, an important element of DNA decondensation [60]. 

The concept of alkaline pH promoting NET formation has been corroborated by the work of other groups as well [61,62]. Behnen et al. noted that PMA-induced NET formation was suppressed by extracellular and intracellular acidification [61]. Inhibited glycolysis and, similar to our findings, ROS production at lower pH was implicated as a potential cause of the observed NET suppression [61]. Maueroder et al. (2016) not only found that alkaline pH promoted NET formation when induced by PMA, ionomycin, and LPS but also that an acidic pH was suppressive [62]. These findings taken together not only emphasize the importance of standardizing pH during experiments but also has implications for the host’s response to bacterial infection during the healing of chronic wounds, which are known to have an alkaline pH [63]. In addition to the various contexts previously discussed, our group has also found that hypertonic saline effectively suppresses Nox-dependent NET formation, with more equivocal effects on Nox-independent NET formation [9]. This suppressive effect was the result of the impairment of ROS production and was reversible through exogenous administration of hydrogen peroxide [9]. The osmolytes d-mannitol and d-sorbitol produced ROS suppression in a similar manner, which is an indication that all three agents may carry out their function through dehydration of neutrophils [9]. 

While NETs have been studied in human patients, murine models of NET formation are also well-validated in the literature and have been used to investigate pathologies ranging from intravascular thrombosis in sepsis to acute lung injury [64,65]. That being said, other models have research utility such as investigating the pro-coagulant status of microcirculatory endothelium in the burn wounds of pigs or the use of glucocorticoids to treat asthma in horses [66,67]. These models are certainly useful for hypothesis generation but their greatest utility comes in the form of evaluating the therapeutic options for treating the plethora of NET-induced pathologies. 

## 6. Clearance of Extruded NETs

Farrera et al. have previously demonstrated that clearance of NETs is dependent on both DNase I and macrophages [68]. In particular, they found that at supraphysiological concentrations of DNase I, NETs were effectively degraded but not at the physiological concentration of 20 ng/mL [68]. Macrophages are physically smaller than NETs and indeed DNase may still play an integral role in clearance as it accelerated the clearance by macrophages, potentially by degrading the DNA backbone into more manageable fragments [68]. Through the administration of cytochalasin, an inhibitor of actin polymerization, and endocytosis inhibitors, such as nystatin, NET clearance via macrophages was shown to be an active process and endocytosis-dependent, respectively [68]. Additionally, Farrera et al. confirmed that macrophages were indeed phagocytosing the NETs, immunoblot assays were carried out on the lysates of these cells and NE was detected in those incubated with NETs but not in macrophage alone group [68]. 

Elucidating the mechanism of NET clearance can potentially play a major role in the development of therapeutics for diseases such as lupus nephritis and acute respiratory distress syndrome (ARDS) [13,69]. Hakkim et al. demonstrated the role of DNase I in the clearance of NETs through its impairment being correlated with kidney pathology in the context of SLE [13]. They found that sera from SLE patients were not as able to effectively degrade NETs as controls, which they hypothesized could be due to the presence of DNase1 inhibitors or obstructive anti-NET antibodies which could prevent substrate binding [13]. Similarly, Gregoire et al. found in ARDS neutrophils that there was not only an exaggerated NET response but also diminished macrophage phagocytosis of NETs [69].

## 7. NET-Induced Pathology

### 7.1. Immunodeficiency to Autoimmunity

As one might expect, NETs play a major role in a variety of immunological diseases ranging from immunodeficiencies such as chronic granulomatous disease (CGD) to autoimmune conditions including systemic lupus erythematosus (SLE). CGD is the result of an inherited deficiency of NADPH oxidase, effectively crippling the ability of phagocytosis and Nox-dependent NET formation to clear both bacterial and fungal infections [70]. This leaves patients particularly vulnerable to aspergillosis, a leading cause of mortality in this population [71]. Conversely, rheumatoid arthritis (RA) is a chronic autoimmune condition characterized by inflammation of synovial joints leading to pain, stiffness, and eventually loss of function [72]. It has been previously demonstrated that NETs isolated from patients with RA had significantly higher spontaneous NET production compared to controls, as well as elevated levels of ROS, NE, MPO, and nuclear translocation of PAD4 [73,74]. Moreover, MPO-DNA complexes, which have been identified as NET remnants of investigative utility, are found at higher levels in patients with RA compared to controls and are associated with increased NET formation and positivity for rheumatoid factor [72]. 

### 7.2. Diabetes and Cardiovascular Disease

Diabetic foot ulcers are among the most disabling and deadly complications of diabetes mellitus so the finding that PAD4 and citrullinated histones are elevated in these wounds is of significant clinical relevance [75,76]. NE, histones, and other NET components were found at elevated levels in the blood of diabetic patients, NE particularly predicting delayed healing [76]. Interestingly, both wild type and diabetic mice experienced accelerated diabetic foot ulcer healing upon administration of DNase [75]. It has also been shown that NET formation can be induced by cholesterol crystals in a Nox-dependent but PAD4-independent manner and NETs have been detected in both mouse and human atherosclerotic lesions [10,77]. A proposed mechanism through which NETs promote inflammation in this context is by upregulating macrophage production of Il-1β as well as indirectly activating TH17 cells [10]. Indeed, biomarkers indicating the presence of NET formation have shown an independent association with atherosclerosis of coronary vessels and severe coronary artery disease [78].

### 7.3. Cancer and Cystic Fibrosis

In recent years, proteins that decorate NETs, such as NE and matrix metalloproteinase-9 (MMP-9) have been investigated for their role in promoting local tumor growth. NE has been shown to enter tumor cells and hyperactivate phosphatidylinositol-3 kinase while MMP-9 clears surrounding extracellular matrix to enable growth [79,80]. NETs have also been shown to promote metastasis by serving as a capture framework for circulating tumor cells. For example, lung carcinoma has a four to five-fold increased adhesion to NETs compared to a neutrophil monolayer [11]. Mutations to the CFTR gene in CF increases the susceptibility of these patients to airway infections, primary among them *Pseudomonas aeruginosa*. Neutrophils are recruited to the airway upon infection and exacerbate the disease by producing NETs which can increase the viscosity of the already problematic mucus in CF patients and enzymes such as NE and MPO can damage respiratory epithelium and connective tissue [81,82]. 

## 8. Potential Pharmacological Manipulations of NET Formation and Extruded NETs

### 8.1. Manipulation of NET Formation

As has been discussed, disruption of physiological NET function can result in pathology and so manipulation of NET formation mechanisms has logically been the target of investigation for the development of therapeutics. In the case of CGD, gene therapy has been shown to restore NET formation through the recovery of Nox and that this treatment was able to resolve refractory invasive pulmonary aspergillosis has tremendous therapeutic implications [71]. However, the bulk of NET related pathology are associated with abundance, rather than a lack of NETs. To this end, diphenyleneiodonium (DPI) has been investigated for its ability to impair NET formation through the inhibition of Nox with success [83]. However, DPI has limited utility as a treatment option because not only does it cause morphological changes in neutrophils, hindering their ability to carry out their antimicrobial function, but also would only be effective in targeting Nox-dependent NET formation [83].

Our group’s work with inhibitors of transcription offers a potential alternative given that this strategy is able to inhibit both Nox-dependent and Nox-independent NET formation while maintaining ROS production so that physiological neutrophil function is not lost [1]. In addition to this, Sollberger et al. (2018) have demonstrated that NET formation is dependent on gasdermin D (GSDMD), a pore-forming protein capable of puncturing granules to release NE [12]. They further identified that a class of molecules based on the pyrazolo-oxazepine scaffold are capable of inhibiting GSDMD and therefore NET formation [12]. In particular, LDC7559 was able to inhibit PMA-induced NET formation but not the physiological function of Nox, NE, or MPO and did not interfere with phagocytosis [12]. Therefore, the identification of a NET formation inhibitor that selectively impairs NET production while sparing phagocytosis and degranulation is a promising avenue of research to be explored [1]. 

### 8.2. Manipulation of Extruded NETs

Administration of DNase to degrade the backbone of NETs is perhaps the oldest therapeutic intervention associated with NET research and it remains one of the most attractive. The locus of interference for DNase follows NET formation and only affects the product of this process. This, in effect, can minimize the effects of NETs specifically in a pathological process without affecting the ability of neutrophils not stimulated into NET formation to phagocytose and degranulate, thus maintaining innate immunity. This is supported by the notion that impaired NET clearance due to the presence of DNase inhibitors or anti-nuclear antibodies preventing access by DNase is associated with pathology in lupus nephritis [13]. It should also be noted that DNase eliminating DNA in the airways of patients with CF has already been demonstrated to have a clinically significant improvement [84]. 

While DNase may seem like an attractive therapeutic option, its use should be critically assessed. Ultimately, DNase assists phagocytes in clearing NETs by fragmenting them and this process may not effectively clear histones or proteases from the vasculature leaving patients open to tissue and organ damage [85]. Additionally, in gout, NETs have been shown to contain and degrade pro-inflammatory mediators thus keeping this effect local [86]. While NETs have been shown to serve as adhesion frameworks for circulating tumor cells, degrading them indiscriminately can leave patients open to systemic inflammation due to the inappropriate liberation of proinflammatory mediators [87]. In short, preventing pathological NET formation, if done in a manner that spares physiological anti-microbial function, should be the focus of research moving forward but DNase is an important therapeutic option to consider. 

## 9. Conclusions

Though the field of NET research is relatively new, key breakthroughs in recent years have illuminated the path towards a unified model of NET formation. The identification of novel forms of NET formation, such as vital NET formation, and refinement of the established Nox-dependent and independent pathways have raised as many questions as they have answered [1,26]. Previously considered unelucidated features of neutrophil biology, such as transcription and mitochondria, have taken on an entirely new relevance [1]. The nuances of NET formation in various physiological contexts including under bloodstream, tissue, alkaline pH, and hypertonic conditions are just beginning to be explored [7,8,9]. The list of serious diseases for which NETs are implicated in the pathophysiology continues to expand and ranges from autoimmune disorders to diabetes to cancer [11,13,75]. These new findings underscore the importance of developing novel therapeutics, which target the process of NET formation and NETs themselves while sparing the crucial role of neutrophils in innate immunity.
